# Anti-predator behavioral responses of Italian agile frog tadpoles (*Rana latastei*) exposed to microplastics

**DOI:** 10.1007/s11356-022-23131-4

**Published:** 2022-09-22

**Authors:** Giovanni Scribano, Andrea Gazzola, Anna Winkler, Alessandro Balestrieri, Alice Grioni, Giuditta Lastrico, Paolo Tremolada, Daniele Pellitteri-Rosa

**Affiliations:** 1 Department of Earth and Environmental Sciences, University of Pavia, I-27100 Pavia, Italy; 2 Department of Environmental Science and Policy, University of Milan, I-20133, Via Celoria, 26 Milan, Italy

**Keywords:** Microplastics, Plastic polymers, Pollution, Anti-predatory response, Tadpoles, Italian Agile frog

## Abstract

Microplastics (MPs) are nowadays abundant, persistent, and ubiquitous in the environment, representing a new threat for terrestrial, marine, and freshwater ecosystems. Although anuran populations and species are globally declining, the effect of MP exposure on this taxon has been poorly investigated. With the aim of assessing the effects of microplastic exposure on the defensive responses of Italian agile frog (*Rana latastei*) tadpoles, we exposed them to three different concentrations (1, 7, and 50 mg L^−1^) of a mixture of plastic polymers (HPDE, PVC, PS, and PES) for 2 weeks. Then, we measured the total distance covered by individual tadpoles before and after exposure to tadpole-fed dragonfly larvae (*Aeshna cyanea*) cues. As expected, predation risk sharply lowered the total distance travelled by tadpoles; however, MP concentration did not affect their defensive performances. We also collected data on tadpole development, activity, and mortality. In contrast with previous experiments, neither tadpole growth nor mortality varied with MP concentration. Our results indicate that the intensity of MP effects on growth and development may depend on tadpole size, with large tadpoles being less susceptible to the negative effects of MP exposure.

## Introduction

In the last decades, thousands of metric tons of plastic waste have ended up in the environment (Hoellein et al. 2014; Geyer et al. 2017) because of the constantly increasing human demand for these polymers. Production increase, coupled with the improvement of plastics’ chemical and mechanical resistance, has made these contaminants more persistent and potentially more hazardous to the environment (Lithner et al. 2011). Microplastic particles (MPs, plastic particles <5 mm in size) are commonly used as raw materials in plastic industries and are contained in several everyday consumer products (e.g., cleaning supplies, toothpaste, synthetic clothes); therefore, these contaminants may be spread in the environment through industrial and domestic wastewaters (Ross et al. 2021). Moreover, MPs can originate from the mechanical and biological fragmentation of plastic waste. Recently, MP contamination has become one of the most addressed forms of environmental pollution (Andrady 2017; Chae and An 2018; Li et al. 2018). MP abundance, persistence, ubiquity, and small size (Hartmann et al. 2019) put these contaminants among the most threatening for a wide range of plant and animal organisms (Anbumani and Kakkar 2018). However, the ecotoxicological effects of MPs on anuran amphibians, one of the most threatened taxa globally (Stuart et al. 2004; Becker et al. 2007), are still lagging behind, with only a few recent studies highlighting their effects on tadpole physiology and behavior (De Felice et al. 2018; Boyero et al. 2020; da Costa Araújo et al. 2020a, b; Balestrieri et al. 2022). Furthermore, studies on the impact of MPs on anuran species of major conservation interest are still lacking.

Anuran tadpoles are suspensivore/grazer primary consumers and are therefore extremely likely to ingest MPs while feeding (Altig et al. 2007; Boyero et al. 2020). Ingestion and accumulation of MPs have been proven in tadpoles both under laboratory and natural conditions (Hu et al. 2016; De Felice et al. 2018; Hu et al. 2018; Karaoğlu and Gül 2020; Kolenda et al. 2020; Balestrieri et al. 2022). Although some studies have shown that tadpoles are capable of tolerating and expelling MPs relatively fast (Hu et al. 2016; De Felice et al. 2018), significant physiological alterations and high mortality levels have been recorded in tadpoles of *Xenopus laevis* and *Alytes obstetricans* (Tussellino et al. 2015; Boyero et al. 2020). Exposure to polyethylene (PE) MPs has been proven to cause histopathological damage in Cuvier’s foam froglet (*Physalaemus cuvieri*) tadpoles (da Costa Araújo et al. 2020a), as well as mutagenic and cytotoxic effects (da Costa Araújo et al. 2020b). Finally, behavioral alterations such as locomotion issues and defective anti-predator defensive response have been observed in tadpoles exposed to PE MPs (da Costa Araújo and Malafaia 2020).

Along with the global decline of amphibian species, there are two main reasons why the effects of MPs on amphibians should be carefully addressed. First, tadpoles are primary consumers in many freshwater ecosystems, and their feeding activity may influence key processes such as primary production or nutrient cycling (Seale 1980; Whiles et al. 2013). Secondly, amphibian larvae and adults may represent an important transfer path for these contaminants through higher trophic levels and between freshwater and terrestrial ecosystems (Larsen et al. 2016; da Costa Araújo and Malafaia 2021).

The Italian agile frog (*Rana latastei*, Boulenger 1879) is an endangered endemic species occurring in northern Italy, Canton Ticino, Istria, Slovenia, and Croatia (Barbieri et al. 2006) in highly fragmented populations. The main threat to this species is the loss of natural habitat caused by urbanization and intensive agriculture, while non-native predator fish and crayfish may have caused the extinction of subpopulations. For these reasons, the Italian agile frog is included in the Annexes II and IV of the Habitats Directive (EC 43/1992) and filed as “Vulnerable” in the IUCN Red List.

Recently, lowered activity levels and development and high mortality rates have been observed in Italian agile frog tadpoles exposed to MPs during early developmental stages (Balestrieri et al. 2022).

Although exposure to anthropogenic pollutants has been shown to affect tadpole behavior in several ways (Rohr and Crumrine 2005; Lavorato et al. 2013; Polo-Cavia et al. 2016; Sievers et al. 2018; Bolis et al. 2020), the effects of MPs on defensive responses have been poorly investigated. Behavioral alterations (e.g., reduced activity, defective anti-predator responses) induced by MPs may play an important role in the decline of anuran population, especially those already threatened by other anthropogenic alterations, such as habitat loss and fragmentation, and alien species.

With the aim of assessing the effects of MPs on the anti-predator responses of Italian agile frog larvae, we exposed 480 tadpoles to three concentrations of a MP mix composed of polyester (PES), polystyrene (PS), polyethylene (HDPE), and polyvinyl chloride (PVC). We predicted the impact of MP exposure to be proportional to MP concentration. Considering the little available information regarding tadpole anti-predator behavior (da Costa Araújo and Malafaia 2020), after MP exposure, we did not expect any unidirectional effect on the intensity of the defensive response. Secondarily, we recorded survival and weight at the end of the experiment, and activity levels during the conditioning period. In this case, we expected a MP concentration-dependent reduction in all variables with respect to controls (Balestrieri et al. 2022).

## Materials and methods

### Animal collection and husbandry

In February 2021, we collected 20 fragments of Italian agile frog egg clutches from three ponds located in a natural protected area (Bosco del Vignolo, 45° 13’ N, 8° 56’ E; Lombardy, N Italy), characterized by several springs, canals, and high forest cover. Water depth was less than 1 m with moderate turbidity and low (<10%) aquatic vegetation cover. Animal collection, husbandry, and testing were authorized by the Ministry of Environment (ISPRA Prot. 1790, 18/01/2021).

Egg clutch fragments were immediately brought to the laboratory and kept in ten, 21-L rearing tanks (2 clutch fragments per tank) filled with dechlorinated tap water until hatching. All tanks were placed in an unheated room under natural light conditions. Mean water temperature ± SD was 20.4 ± 0.7°C throughout the study period. Ten late instar dragonfly larvae (*Aeshna cyanea*) were collected using dip nets from ponds located into the Botanical Garden of Pavia. Predators were individually kept in 0.8-L tubs filled with 0.5 L of dechlorinated tap water. A small piece of mesh was provided as perching site in each tub.

### Production of MPs

Following the procedure described in Balestrieri et al. (2022), we prepared a MP mix consisting of polyvinyl chloride (PVC) from orange pipes, high-density polyethylene (HDPE) from red bottle caps, polyester fibres (PES) from blue-coloured synthetic fabrics, and expanded polystyrene (PS) from black foam food trays. Polymers were then mixed in three concentrations: 1, 7, and 50 mg L^−1^. The lowest tested concentration was consistent with mean concentrations at the outlet of wastewater treatment plants (6400 MP m^−3^, Schmidt et al. 2020; assuming spherical MPs with a mean diameter of 700 μm and a density of 1 g cm^−3^, 6400 MP m^−3^ correspond to 1.15 mg L^−1^). The other two concentrations (7 and 50 mg L^−1^) followed a geometrical increase and represented the worst-case scenarios based on the highest concentration (450 000 MP m^−3^) reported by Schmidt et al. (2020). To prevent low-density polymer particles from being quantitatively overrepresented, MPs were weighed using a high-precision scale to obtain a constant weight-ratio of 3 PVC : 3 HDPE : 3 PES : 1 PS. All tested concentrations were environmentally relevant (Schmidt et al. 2020) and were lower than those previously used for assessing the effects of MP exposure on anuran tadpoles (da Costa Araújo et al. 2020a).

### Experimental procedure

Two weeks after hatching, a subsample of tadpoles (*N* = 20, which were excluded from trials) was staged following Gosner (1960) and wet-weighted (mean stage ± SE= 28 ± 0.15; mean weight ± SE = 57 ± 2.6 mg). A total of 480 tadpoles was then selected for the experiment (120 tadpoles per treatment and 120 as controls). Tadpoles were distributed into 24 tanks filled with 8 L of dechlorinated tap water (31.5 × 22.5 × 25 cm; 20 tadpoles per tank, two from each of the 10 rearing tanks), which were grouped into 6 blocks, each including all MP treatment levels (1, 7, 50 mg L^−1^) and a control tank (0 mg L^-1^). Within each block, treatments were randomly assigned to tanks. All tanks were checked for dead tadpoles twice a day (at 9 a.m. and 6 p.m.), and a standardized quantity of rabbit chow (170 mg, i.e., ca. 15% of the wet mass of 20 tadpoles at the start of the trial) was provided daily. At the end of the experimental period (when tadpoles were 30 days old), ten randomly chosen tadpoles from each tank (240 in total) were wet weighted with a high precision scale (± 0.01 mg).

### Tadpole behavior

#### Activity

To assess the activity level of tadpoles belonging to different treatments, we recorded the percentage of active tadpoles (i.e., swimming or foraging) during five 10-min sessions at day 3, 5, and 7 of exposure, twice from 9 to 10 am and three times from 3 to 4 pm. All sessions were video recorded using a digital camera (Olympus Tough TG-5), hung up 1 m above the testing tanks. Tanks belonging to the same block were recorded simultaneously. The number of active tadpoles was assessed in a 10-s interval within each minute, comparing consecutive 1-s frames and counting the number of individuals which changed their position inside the tank at each 1-s interval. A total of 2000 observation were made for each tank (20 tadpoles × 10 1s intervals × 10 min) and the activity level was assessed as (total N of movements / 2000) × 100. Frame to frame movements shorter than tadpole body depth and rotations were excluded from the analysis. The observer was blind with respect to the treatment assigned to each experimental tank.

#### Defensive response

To obtain olfactory cues for anti-predator tests, dragonfly larvae were fed with Italian agile frog tadpoles. Each predator was provided with the same prey weight (usually two tadpoles, ≈ 130 mg). Before the beginning of every trial (1 h after feeding), we collected 10 ml of water from 5 randomly selected predator tubs. Aliquots were mixed in the same container and 2 ml of the resulting mixture was then used as odor cues for anti-predatory trials. Every day, predator tubs were carefully washed and refilled to keep the water volume constant and prevent signal contamination.

To test for tadpole anti-predator responses after 2 weeks of exposure to MPs, 36 tadpoles per treatment (6 tadpoles per tank) were individually moved into white plastic arenas (15 × 10.5 cm) filled with 250 ml of dechlorinated tap water and left to acclimatize for 15 min. Arenas were shielded by opaque panels and uniformly lightened by spotlights. All trials included a 15-min pre-stimulus (before cue infusion) and a 15-min post-stimulus (after cue infusion) video recording periods, which were recorded using a digital camera (Canon Legria) hung up 1.2 m above the arenas. Each trial included 12 arenas, in which tadpoles from different treatment levels were randomly distributed. To minimize disturbance, the stimuli, either 2 ml of predator cues or water (control), were gently injected with a 10-ml disposable syringe. A total of 144 tadpoles were tested (18 tadpoles × 2 cues × 4 MP treatments) in 2 days (6 trials per day, between 9 a.m. and 14 p.m.). The concentration of predator cues used for behavioral trials (1:125) was consistent with previous studies (e.g., Gazzola et al. 2018, 2021; Scribano et al. 2020).

All video clips were analyzed using ToxTrac (Rodriguez et al. 2018), which provides locomotor information by recording the *x* and *y* coordinates of the central point of each tadpole every 0.04 s. We used the locomotor variable “total distance,” namely the total distance (mm) covered by each tadpole during the trial (pre- and post-stimulus), as an index of tadpole activity level.

### Statistical analysis

To analyze the effects of MP exposure on tadpole behavior (i.e., activity levels recorded within the experimental containers), we ran a linear mixed model (LMM) with the proportion of active tadpoles as response variable. MP treatment, recording session (factor with five levels), and their interaction were included as fixed effects, block as random effect.

Tadpole weights at the end of the experiment were explored using a LMM, with the mean tadpole mass recorded for each tank (experimental unit) as response variable. Treatment and block were included as fixed and random effects, respectively.

Tadpole behavior during anti-predatory tests was also explored by a LMM, using the proportional change in total distance [pctd = (post-stimulus – pre-stimulus) / pre-stimulus)] as response variable. Tank within block was included as random factor, and predator cue (factor with two levels), MP treatment and their interaction as fixed factors. We used the varIdent function (implemented in R package *nlme*) to account for unequal variances between predator treatments. The same method was used to explore tadpole total distance before stimulus injection (i.e., in absence of predatory cues), with MP treatment as fixed effect.

LMMs were run using the R package *lme4* (Bates et al. 2015) and *nlme* (Pinheiro et al. 2022). The estimated means and planned comparisons among MP treatments and predator cues were obtained using the *emmeans* package (Lenth 2022). The “Anova” function of R package *car* was used for the analysis of deviance tables (Fox and Weisberg 2019), reporting Wald chi-squared tests. All model assumptions were explored by checking residual distribution against fitted values (Tukey–Anscombe plot) and residual normality against the theoretical normal distribution (quantile-quantile plot).

## Results

**Fig. 1:**
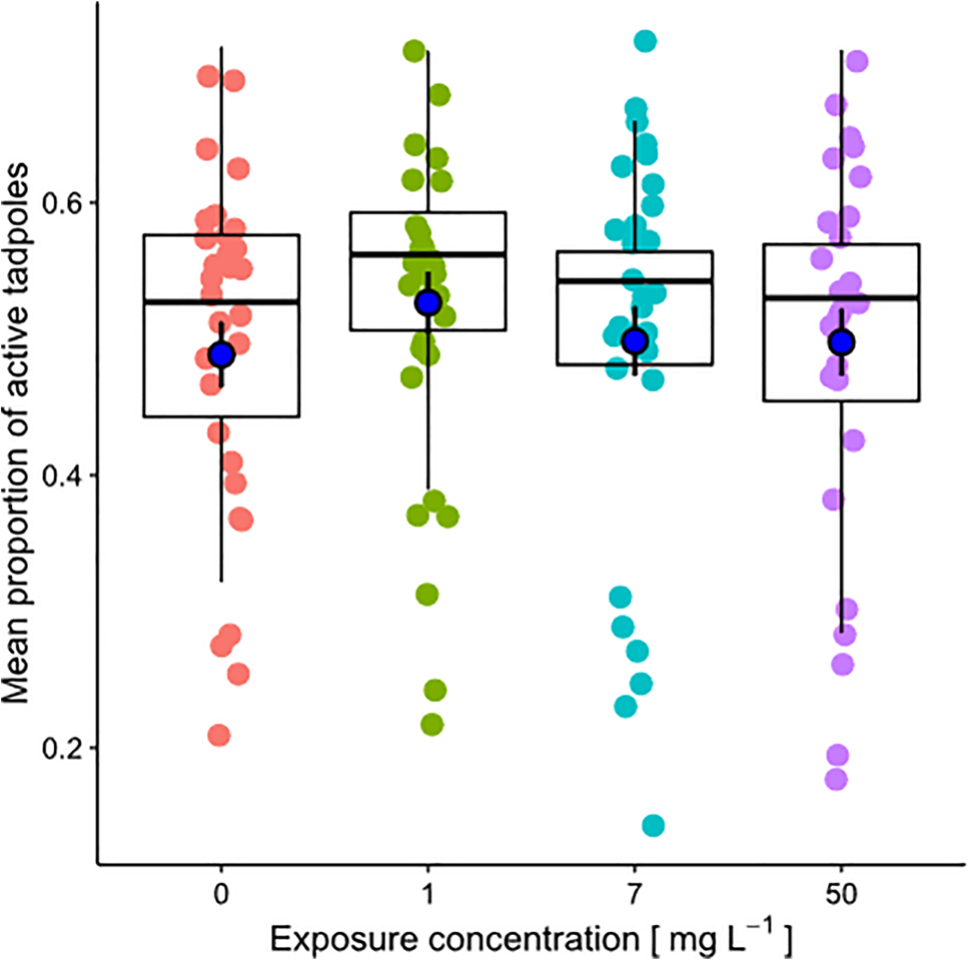
Mean proportion of active tadpoles (blue dots represents grand means ± SE) recorded during the entire conditioning period of the experiment for each of the four MP concentrations (0, 1, 7, 50 mg L^−1^). Each colored dot represents the mean proportion of active tadpoles per tank, during the five periods of recording (*N* = 30 for each MP concentration); box plots show medians (lines in the boxes), 25% and 75% quartiles (boxes)

Tadpole activity was not affected by treatment (*χ*^2^ = 6.71, df = 3, *P* = 0.08), while was highly influenced by the recording session (*χ*^2^ = 439.15, df = 4, *P* < 0.0001). The interaction between these factors was not significant (*χ*^2^ = 7.83, df = 12, *P* = 0.80). Moreover, during all recording sessions, no significant difference was detected between each treatment and the respective control (lower *P* = 0.31; Fig. 1).

**Fig. 2:**
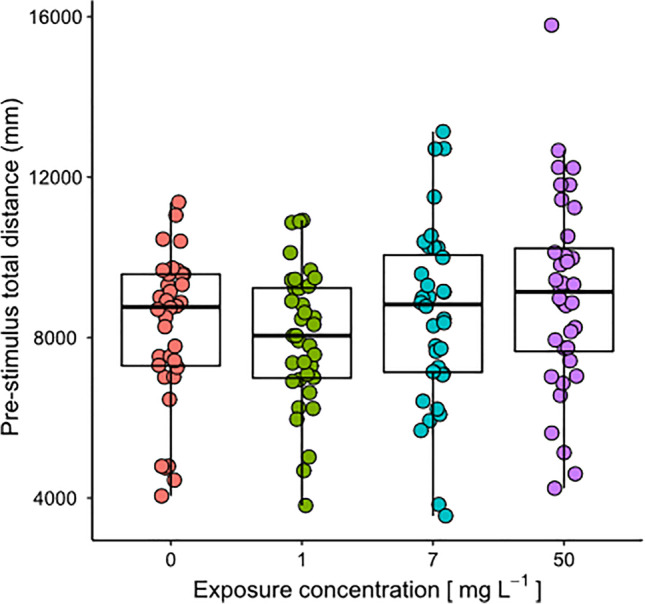
Total distance covered by tadpoles before the stimulus injection during the anti-predatory experiment for each of the four MP concentrations (0, 1, 7, 50 mg L^−1^). Colored dots represent individual data (*N* = 36 for each MP concentration); box plots show medians (lines in the boxes), 25% and 75% quartiles (boxes)

**Fig. 3:**
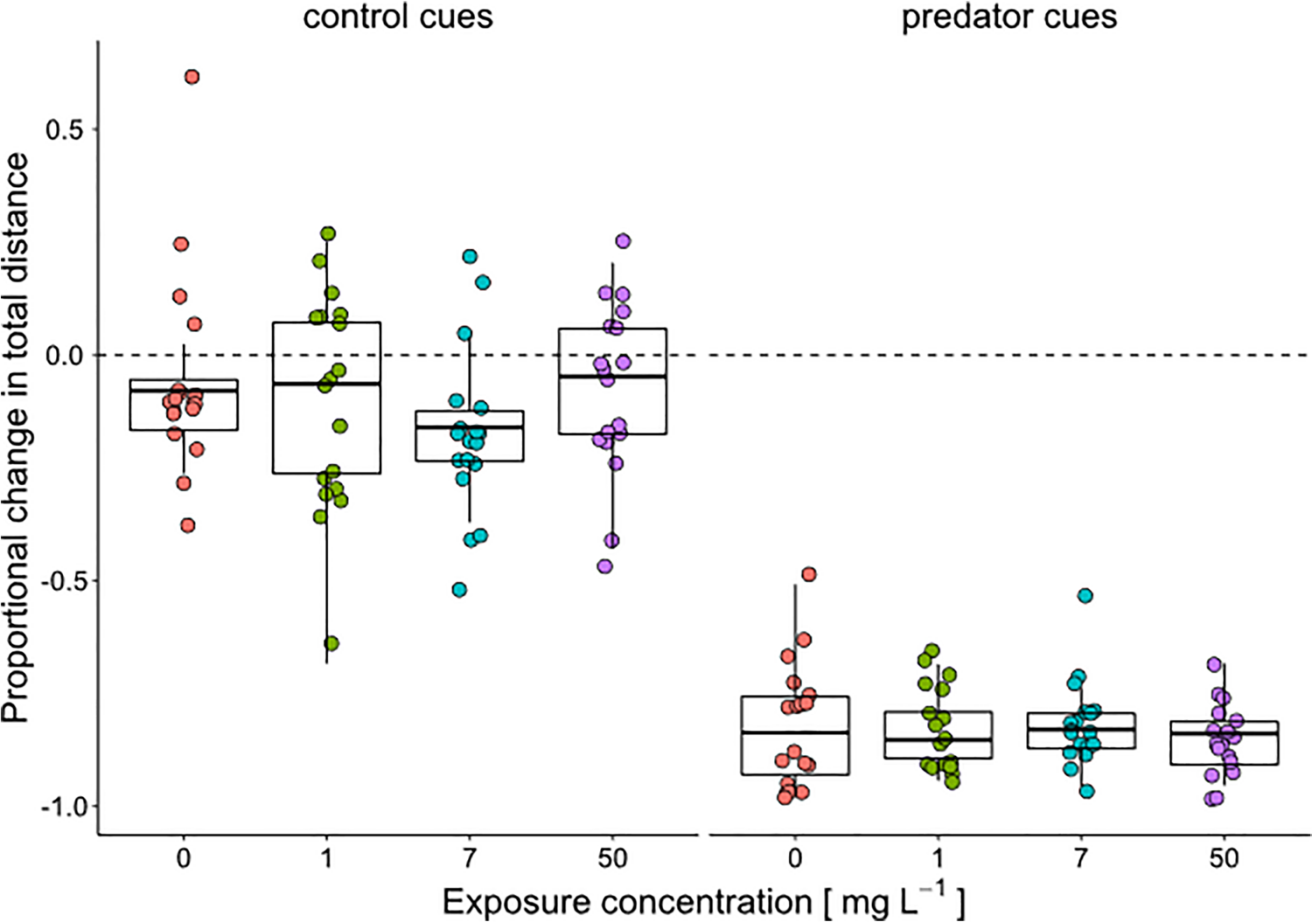
Variation in total distance covered by tadpoles after the injection of the stimulus (water as control or predator cue) respect to the pre-stimulus baseline for each of the four MP concentrations (0, 1, 7, 50 mg L^−1^). Colored dots represent individual data (*N* = 18, for each MP concentration); box plots show medians (lines in the boxes), 25% and 75% quartiles (boxes). The dashed line indicates a reference point for equal pre- and post-stimulus distance

During the anti-predator experiment, the total distance covered by tadpoles before stimulus injection was not affected by MP treatment (*χ*^2^ = 5.86, df = 3, *P* = 0.12, Fig. 2), although a nearly significant increase was observed when comparing the highest MP concentration and controls (estimated difference: 0–50 = −845± 476, df = 141, t.ratio = −1.77, *P* = 0.07). Tadpoles belonging to all treatments responded to predator cues by strongly decreasing their total travelled distance respect to controls (water injection; Fig. 3). Proportional change in total distance (pctd) was significantly affected by predator cue (*χ*^2^ = 810.11, df = 1, *P* < 0.0001), but neither by MP treatment (*χ*^2^ = 2.89, df = 3, *P* = 0.40) nor the interaction between cue and treatment (*χ*^2^ = 2.99, df = 3, *P* = 0.39). Planned contrasts showed that pctd of predation cue-exposed tadpoles was significantly lower than the respective control (water) for all microplastic treatments (lowest estimated difference = 0.66 ± 0.05, df = 116, t-ratio = 12.79, *P* < 0.0001; Fig. 3). However, pctd of tadpoles exposed to predator cues did not differ between any MP treatment and the control (0 mg L^−1^) (highest estimated difference = 0.03, df = 131, *P* = 0.52), that is all five treatments showed a similar defensive behavior.

**Fig. 4:**
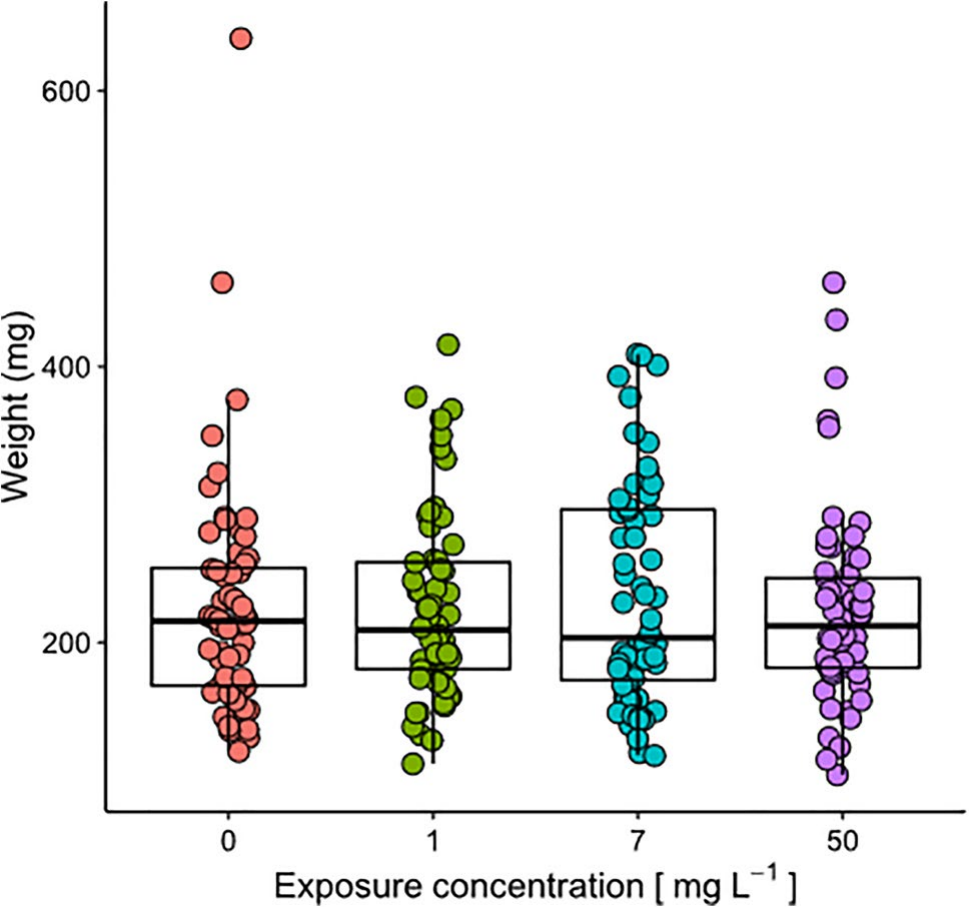
Tadpole weights at the end of the experiment for each of the four MP concentrations (0, 1, 7, 50 mg L^−1^). Colored dots represent individual data (*N* = 60 for each MP concentration); box plots show medians (lines in the boxes), 25% and 75% quartiles (boxes)

Mortality rate at the end of the experimental period was null for all treatments. Tadpole mass (mean ± SE final weights: control = 227.3 ± 10.8 mg; 1 mg L^-1^ = 225.2 ± 8.7 mg; 7 mg L^-1^ =234.2 ± 10.4 mg; 50 mg L^-1^ = 222.6 ± 9 mg) was not affected by long term exposure to microplastic treatments (*χ*^2^ = 2.20, df = 3, *P* = 0.53), and no difference was observed for any treatment respect to controls (lower *P* = 0.12; Fig. 4).

## Discussion

Unexpectedly, MP exposure affected neither tadpole defensive behavior, nor activity levels and growth. The chemical cues of tadpole-fed dragonfly larvae sharply lowered the total distance travelled by tadpoles. A reduction in activity levels is a widespread anti-predator response of prey species threatened by conspecific-fed, native predators, with which they can be assumed to share a long history of co-evolution (Schoeppner and Relyea 2009; Hettyey et al. 2015; Gazzola et al. 2018).

Contrary to the tadpoles of Cuvier’s foam froglet (da Costa Araújo and Malafaia 2020), exposure to MPs had no effect on the performance of Italian agile frog tadpoles during anti-predatory trials. As the developmental stage of tadpoles was similar for both studies, conflicting results may depend on MP concentration, which was higher in the previous study (60 mg L^−1^), or tadpole size, Cuvier’s froglet larvae being smaller than Italian agile frog’s (139 ± 47 mg vs. > 222 mg, respectively, at the end of the experiments), and thus possibly less efficient in egesting MPs. Moreover, the defensive response of froglet tadpoles was assessed using an index of social aggregation, with the aim of testing the anxiogenic effect of MPs, which is why we cannot exclude contradictory results to have been provided by differing experimental protocols (i.e., testing of single individuals vs. groups of 8 tadpoles) or targeted stressor mechanisms (i.e., the choice of which type of response to measure). Another possible explanation might be intrinsic to experimental methods; for example, the concentration of predator odor might have been too high to reveal reliable behavioral differences among MP treatments. Moreover, the behavioral variable we collected might have been not ideal for revealing the potential effects of MP exposure, or again, potential differences were not observable by merely exploring behavioral (Gazzola et al. 2015) or life history traits.

The lack of changes in the behavioral defensive responses agrees with the recording of no effects of MP exposure on tadpole growth and activity levels and suggests that either agile frog tadpoles could avoid MP ingestion or efficiently egested them. Based on our previous knowledge on MP exposed tadpoles of the same species, these results were unexpected, as, when tested in an slightly earlier stage of development, tadpoles showed low activity levels, arrested development, and concentration-dependent, high mortality rates (Balestrieri et al. 2022).

Two hypotheses can be made to explain these differences. First, as captivity conditions can affect development (Matson et al. 2010; Mendelson III and Altig 2016), tadpole size may be a more effective parameter to compare the two experiments. At the beginning of our experiment tadpoles were by far larger (57 ± 1.2 mg vs. 20 ± 1 mg) than those tested by Balestrieri et al. (2022), suggesting that, as hypothesized for Cuvier’s foam froglet (da Costa Araújo and Malafaia 2020), tadpole size may have played a major role in driving the effects of MP exposure (see also De Felice et al. 2018 about *Xenopus laevis* and *X. tropicalis*).

Secondly, different from the first experiment, which was carried out on 1-week-old larvae naïve to both rabbit chow and MPs, we fed tadpoles for ten days before the beginning of the trials. Since the same food was provided during trials, it is possible that habituation to food enhanced the avoidance of MPs. Since *Rana* tadpoles are opportunistic feeders which usually ingest a wide variety of edible and non-edible particles (Pozzi 1980; Altig et al. 2007; Lanza et al. 2007), further studies are needed to understand if they can select specific food resources.

The recorded lack of MP effects of growth and activity is consistent with the findings of De Felice et al. (2018), who tested *Xenopus laevis* embryos at MP concentrations ranging between 0.125 and 12.5 mg L^−1^, suggesting that also species-specific differences may account for discrepancies in the results.

In response to the high level of threat affecting amphibians (Stuart et al. 2004; Beebee and Griffiths 2005), translocations have sometimes been carried out to enhance the recolonization of suitable areas (Denton et al. 1997; Fisher 1999; Sarrazin and Legendre 2000; Thompson et al. 2022). Nonetheless, considering the low success rate of reintroduction attempts (e.g., for *R. latastei*: Scali et al. 2001; Bernini and Razzetti 2002; Pellitteri-Rosa et al. 2008), several authors have questioned the effectiveness of these practices (Burke 1991; Dodd and Seigel 1991; Moritz 1999).

The quality of released individuals is a major factor to consider in any amphibian reintroduction or relocation program (Mendelson III and Altig 2016). Usually, the introduction of egg masses or early life stages is preferred, both for testing the suitability of the ponds for larval development and reducing the impact on donor populations (Buckley and Foster 2005). While translocation success has been reported to be independent from life stage (Germano and Bishop 2009), our results suggest that the size of released tadpoles may affect their probability of survival in waters contaminated by MPs.

## Conclusions

Despite the growing number of studies investigating the effects of microplastics on a wide variety of organisms, their ecotoxicological effects on anuran amphibians have been poorly addressed, as so as those on behavioral responses. Moreover, anuran larvae may act as an entry for MPs in trophic webs and may prime bioaccumulation in higher trophic levels. Accordingly, da Costa Araújo and Malafaia (2021) recorded the transfer of MPs through an experimental food chain including *Physalemus cuvieri* tadpoles, fish, and Swiss mice; in a short time, MPs were transferred along the food chain affecting the activity and anti-predator responses of the highest trophic level (mice). Up to now, the effects of MPs on behavior have been poorly investigated, leading to contrasting results, possibly depending on laboratory protocols and interspecific variation in susceptibility. In the case of anuran larvae, although the effects of MPs are far to be elucidated, our results indicate that tadpole size, either depending on intra- or inter-specific differences, and/or feeding habituation may reduce the negative effects of MPs in polluted environments. Whether size or the length of the larval stage may also shape interspecific variation in bioaccumulation levels is worth of further investigations.

## Data Availability

Data presented in this study were stored electronically and they are available on request from the corresponding author.
